# Reviewed article: Chaves ACKA, Meucci RD. Monitoring influenza vaccination coverage among older adults: a rural cohort study, Rio Grande, 2017-2022. Epidemiol Serv Saude. 2026:35;e20250561.

**DOI:** 10.1590/S2237-96222026v35e20250561.c

**Published:** 2026-03-23

**Authors:** Mariana Del Grossi Moura

**Affiliations:** 1 Instituto Sírio-Libanês de Ensino e Pesquisa, São Paulo, SP, Brazil

## First round comments

Após a revisão do seu artigo, identificamos a necessidade de uma revisão substancial para que o manuscrito esteja de acordo com os padrões editoriais da revista e para viabilizar sua publicação.

Além dos pontos detalhados pelos revisores, reforço a necessidade de atenção especial aos seguintes aspectos:

• Método: não está claro se a análise foi realizada com todos os participantes em cada ponto de coleta ou apenas com os 651 idosos que participaram dos três momentos do estudo. Solicita-se explicitar o método de amostragem adotado e, se aplicável, o plano de amostragem e o cálculo do tamanho amostral. A construção da variável “três doses” também carece de detalhamento: é necessário esclarecer que se trata de uma variável ordinal derivada da soma das respostas afirmativas à pergunta sobre vacinação nos três anos avaliados, interpretada como uma trajetória de adesão vacinal. Recomenda-se que a seção de métodos seja revisada à luz do checklist STROBE, a fim de assegurar maior transparência e completude na descrição do delineamento, das variáveis e das análises.

• Terminologia: revisar o uso do termo “prevalência” no contexto de estudo de coorte. Recomenda-se substituí-lo por expressões como “cobertura vacinal” ou “proporção de indivíduos vacinados”, que são mais apropriadas conceitualmente ao tipo de análise realizada; revisar o termo “idoso”, utilizar “pessoa idosa”, para alinhamento de linguagem inclusiva e respeitosa

• Discussão: recomenda-se aprofundar a análise das limitações do estudo, incluindo:

- Os possíveis efeitos do viés de atrito, considerando que os participantes que permaneceram no estudo podem diferir sistematicamente dos que foram perdidos ao longo do seguimento (ex: maior engajamento com os serviços de saúde ou melhor estado de saúde geral);

- A ausência de validação do desfecho autorreferido, uma vez que a vacinação foi aferida por autorrelato, sem confirmação documental (ex: cartão de vacinação ou prontuário), o que pode gerar viés de memória ou desejabilidade social, especialmente em populações idosas;

- As limitações do modelo estatístico utilizado, em particular no que se refere aos pressupostos da regressão logística ordinal;

- E o reconhecimento das restrições à inferência causal, inerentes a estudos observacionais de base populacional.

• Redação científica: recomenda-se a realização de uma revisão gramatical e textual, visando à correção de trechos com problemas de coesão e coerência, bem como à adequação da terminologia e do estilo à linguagem científica exigida pela RESS.

Solicitamos que todos os comentários da revisão por pares sejam respondidos pontualmente por meio de uma carta resposta detalhada, explicando as modificações realizadas ou justificando, quando necessário, a manutenção do texto original. A nova versão do manuscrito será reconsiderada após essa reformulação e poderá passar por nova rodada de avaliação. Destacamos que esta decisão não garante a aceitação final do manuscrito.

Recommendation: Major revision

